# Analysis of single nucleotide polymorphism of *SYNE1* gene in Oral squamous cell carcinoma

**DOI:** 10.7150/jca.139098

**Published:** 2026-08-24

**Authors:** Ming-Ju Hsieh, Hsin-Yu Ho, Yu-Sheng Lo, Bharath Kumar Velmurugan, Shanmugavadivu Muthusamy, Min-Yun Kao, Chia-Chieh Lin, Yi-Ching Chuang, Mu-Kuan Chen

**Affiliations:** 1 Oral Cancer Research Center, Changhua Christian Hospital, Changhua 50006, Taiwan.; 2 Graduate Institute of Clinical Medicine, College of Medicine, National Chung Hsing University, Taichung 402202, Taiwan.; 3 Graduate Institute of Biomedical Sciences, China Medical University, Taichung 404328, Taiwan.; 4 Department of Dental Technology and Materials Science, Central Taiwan University of Science and Technology, Taichung 406, Taiwan.; 5 Maitocon Laboratory, Tirupur, 641604, India.; 6 Department of Biotechnology, Dr. N. G. P. Arts and Science College, Coimbatore, India.; 7 Department of Otorhinolaryngology, Head and Neck Surgery, Changhua Christian Hospital, Changhua 50006, Taiwan.

**Keywords:** OSCC, SYNE1, polymorphism, rs2295190, betel nut

## Abstract

Oral squamous cell carcinoma (OSCC) is one of the major causes of cancer-related mortality in Taiwan and is strongly associated with alcohol consumption, betel quid chewing, and cigarette smoking. Spectrin repeat-containing nuclear envelope protein 1 (*SYNE1*) is downregulated in various cancer types, but its role in OSCC remains unclear. The aim of the present study is to investigate the association between single nucleotide polymorphisms (SNPs) of *SYNE1* gene (rs2295190, rs2306916, rs2695261, rs9371601) and OSCC susceptibility as well as clinical outcomes in 377 OSCC patients and 480 cancer-free controls. No significant association was found between any of the four SNPs and overall OSCC risk. However, in OSCC patients, *SYNE1* rs2295190 GT+TT genotype was significantly associated with advanced clinical stage (Stage III/IV) compared to the GG genotype (AOR=2.271, 95% CI: 1.081-4.771, *p=0.030*). This association was particularly evident in betel nut chewers (OR=2.399, 95% CI: 1.026-5.609, *p=0.043*) and in those using the areca nut with slaked lime and piper betel leaf (OR=3.482, 95% CI: 1.063-11.408, *p=0.039*). Although no significant association was observed between the four investigated SNPs and overall OSCC susceptibility, the *SYNE1* rs2295190 GT+TT genotype was significantly associated with advanced clinical stage in OSCC patients. This association was more evident among individuals with betel nut chewing habits, mostly in OSCC patients who consumed areca nut with slaked lime and piper betel leaf. These findings suggest that *SYNE1* rs2295190 may be associated with OSCC progression rather than overall disease susceptibility, particularly in the context of betel quid exposure.

## Introduction

Cancer is one of the leading causes of death worldwide 1. In Taiwan, cancer caused 53,000 deaths in 2023, accounting for 25.84 % of all deaths and making it the leading cause of mortality (Ministry of Health and Welfare, Taiwan, ROC, 2023). Oral cavity squamous cell carcinoma (OSCC) is one of the most common cancers worldwide, with almost 377,000 new diagnoses and 177,000 deaths estimated in 2020^2^. Alcohol consumption, betel quid chewing, and cigarette smoking are the primary risk factors for the development of oral cancer 3. OSCC is often diagnosed at later stages (stage III/IV), where treatment is not effective. Thus, identifying genetic biomarkers that predict aggressive tumour behaviour is essential for improving patient outcomes.

The spectrin repeat-containing nuclear envelope protein 1 (SYNE1) gene encodes nesprin-1, a scaffold protein involved in linking the cytoskeleton, nuclear envelope, and other subcellular compartments 4. Dysregulation of *SYNE1* has been associated with several cancers, including colorectal and prostate cancer 5, 6. According to Pengju Li et al 7, *SYNE1* was a potential biomarker associated with high tumor mutation burden (TMB) and improved response to immune checkpoint inhibitors (ICI) in renal cell carcinoma.

However, the role of *SYNE1* genetic polymorphisms in oral cancer progression and clinicopathologic features was not understood. In the present study, we investigated four SYNE1 SNPs (rs2295190, rs2306916, rs2695261, and rs9371601) to determine their associations with OSCC susceptibility and clinicopathological characteristics, with particular consideration of environmental risk factors.

## Materials and Methods

### Patients and Specimens

This study was approved by the Institutional Review Board of Changhua Christian Hospital (CCH; IRB No. 130616). A total of 857 participants were enrolled between 2014 and 2023, including 377 histopathologically confirmed OSCC patients and 480 cancer-free controls. All participants were of Han Chinese ethnicity and provided written informed consent. Demographic data, personal habits (betel nut chewing, smoking, alcohol consumption), and clinical parameters (AJCC 8th edition TNM stage, tumor differentiation) were obtained from medical records. Venous blood was collected in K3-EDTA tubes and stored at -80 °C.

### Functional SYNE1 SNP Selection

Due to limited references, SNPs were selected from the ABI probe database for Asian populations. Linkage disequilibrium (LD) sites (r² > 0.8) and SNPs with minor allele frequency (MAF) < 5% were excluded. Four polymorphisms were selected: rs2295190 (G/T), rs2306916 (T/A), rs2695261 (A/G), and rs9371601 (T/G).

### DNA Extraction and Genotyping

Genomic DNA was extracted from peripheral blood leukocytes using the QIAamp DNA Blood Mini Kit (QIAGEN, Hilden, Germany; Cat. No. 51104) according to the manufacturer's instructions. Genotyping of SYNE1 rs2295190, rs2306916, rs2695261, and rs9371601 was performed using TaqMan SNP Genotyping Assays (Applied Biosystems / Thermo Fisher Scientific, Foster City, CA, USA; Assay IDs: [rs2295190, C_16189468_10], [rs2306916, C_16195037_10], [rs2695261, C_16059446_30], and [rs9371601, C_30272083_10], respectively) with TaqMan Genotyping Master Mix (Applied Biosystems / Thermo Fisher Scientific; Cat. No. 4371355) on an ABI StepOne Real-Time PCR System (Applied Biosystems). Each 5 µL reaction contained 2.5 µL TaqMan Genotyping Master Mix, 0.125 µL probe mix, and 30 ng genomic DNA. The PCR protocol was: 95°C for 10 min, followed by 40 cycles of 95°C for 15 s and 60°C for 1 min.

### Statistical Analysis

Statistical analyses were performed using IBM SPSS v22.0. Differences in continuous variables were assessed using the Mann-Whitney U test, whereas categorical variables were compared using the chi-square test or Fisher's exact test, as appropriate. Adjusted odds ratios (AORs) and 95% confidence intervals (CIs) were calculated using multiple logistic regression models after controlling for betel nut chewing, alcohol consumption, and tobacco smoking. A *p*-value < 0.05 was considered statistically significant.

## Results

### Demographic and Clinical Characteristics

A total of 480 controls and 377 patients with OSCC were included in this study. Betel nut chewing, cigarette smoking, and alcohol consumption were significantly more prevalent among patients with OSCC than among controls (Table 1).

### Association of *SYNE1* SNPs with OSCC Risk

Genotype distributions for all four *SYNE1* SNPs in controls and OSCC patients were listed in Table 2. After adjusting for betel nut, alcohol, and tobacco use, no significant association was found between any SNP and overall OSCC risk. For rs2295190, the GT+TT genotype did not significantly increase OSCC risk (AOR=0.792, 95% CI: 0.457-1.371, *p*=0.404). Similarly, rs2306916 (TA+AA: AOR=0.924, *p*=0.670), rs2695261 (AG+GG: AOR=0.817, *p*=0.292), and rs9371601 (TG+GG: AOR=0.981, *p*=0.910) showed no significant associations.

### Association of *SYNE1* rs2295190 with OSCC characteristics

Although rs2295190 was not associated with OSCC risk, we further analysed its relationship with clinical progression (Table 3). Notably, rs2295190 GT+TT genotype was significantly associated with advanced clinical stage (Stage III/IV). Among patients with the GT+TT genotype, 64.7% presented with Stage III/IV disease, compared to 43.7% of patients with the GG genotype (AOR=2.271, 95% CI: 1.081-4.771, *p*=0.030). No significant associations were observed for tumor size, lymph node metastasis, distant metastasis, or cell differentiation. We further stratified OSCC patients by betel nut chewing status (Table 4). The association between rs2295190 GT+TT and advanced clinical stage was significant only in betel nut chewers (n=253; OR=2.399, 95% CI: 1.026-5.609, *p*=0.043), but not in non-chewers (*p*=0.296). This finding suggests that the association may be more evident among individuals with betel quid exposure.

To further clarify the influence of betel quid composition, subgroup analyses were performed based on the type of betel quid additive used (Table 5). A significant association between the rs2295190 GT+TT genotype and advanced-stage OSCC was observed in individuals who consumed areca fruit together with slaked lime and Piper betel leaf; carriers had a significantly higher likelihood of advanced-stage disease (OR = 3.482, 95% CI: 1.063-11.408, p = 0.039). No statistically significant associations were found in the other additive subgroups. These results suggested that some components of betel quid might further enhance the effect of SYNE1 rs2295190 on the development of OSCC.

## Discussion

In the present study, we examined the correlation of four *SYNE1* polymorphisms with oral squamous cell carcinoma (OSCC) susceptibility and progression in Taiwanese population with predominant betel quid exposure. Although we did not observe a significant association between the *SYNE1* SNPs and overall susceptibility to OSCC, we did observe a significant association between the *SYNE1* rs2295190 GT+TT genotype and advanced clinical stage. The association was mainly observed among betel nut chewers, suggesting that the clinical association of rs2295190 may be more evident in the context of environmental carcinogen exposure.

Betel quid chewing is one of the strongest etiological factors for OSCC in Taiwan, India, and several South Asian populations 8, 9. The International Agency for Research on Cancer (IARC) has classified BQ (without added tobacco) and areca nut as group 1 carcinogens 10. Areca nut constituents include several alkaloids (0.15-0.67%), polyphenols (11-26%), fats (1.3-17%), saccharides (26-47%), and minor amounts of crude fiber and tannins, including gallotannic acid and phlobatannin 8, 11, 12. Arecoline, the principal alkaloid, modulates several cellular enzymes like matrix metalloproteinases (MMPs) and lysyl oxidase—and suppresses p53 mRNA expression as well as DNA repair mechanisms 13-15. In multiple malignancies, including colorectal, gastric, ovarian, and renal cancers, SYNE1 dysregulation has been associated with tumour progression, cell migration, and genomic instability 5, 7, 16, 17.

*SYNE1* expression is epigenetically repressed through hypermethylation of the CpG island, residing in either the promoter or coding region 18, 19.* SYNE1* encodes nesprin-1, a key component of the linker of nucleoskeleton and cytoskeleton (LINC) complex, which maintains nuclear architecture, chromosomal stability, and mechanotransduction^20^. Public functional annotation was further performed to explore whether rs2295190 may influence SYNE1 function. GTEx eQTL analysis did not identify rs2295190 as a significant eQTL for SYNE1, and PancanQTLv2 HNSC analysis did not support rs2295190 as a significant fine-mapped tumor eQTL or GWAS-linked eQTL. However, GTEx sQTL analysis identified three significant associations between rs2295190 and SYNE1 intron usage, suggesting a potential effect on SYNE1 splicing. Together with its nonsynonymous annotation, these findings raise the possibility that rs2295190 may influence SYNE1 through splicing-related or protein-level mechanisms rather than by altering total SYNE1 expression. Chronic exposure to betel quid constituents can induce oxidative stress, DNA damage, and other cellular stresses in oral epithelial cells. Therefore, one possible explanation for our epidemiological observation is that genetic variation in or near SYNE1 may modify cellular responses to chronic betel quid-associated stress, thereby influencing OSCC progression. However, the present study did not directly evaluate SYNE1 expression, alternative splicing, protein function, nuclear integrity, or DNA damage responses according to rs2295190 genotype. Thus, the proposed functional mechanisms remain hypothetical and require validation in future studies.

Interestingly, the strongest association was identified among individuals consuming areca nut together with slaked lime and *Piper betel* leaf. Slaked lime increases the alkalinity of betel quid, enhancing arecoline release and ROS generation, while *Piper betel* leaf may further increase mucosal permeability and carcinogen exposure 21, 22. These findings suggest that specific betel quid compositions may increase the interaction between environmental carcinogens and genetic susceptibility factors such as *SYNE1* rs2295190. The observed variability of betel quid constituents among populations may thus partially explain the differences in OSCC progression patterns in betel quid users.

Interestingly, rs2295190 was not significantly associated with tumour size, lymph node metastasis, distant metastasis or histological differentiation. This suggests that the association of rs2295190 may be more closely related to overall disease stage than to any individual clinicopathological parameter. To our knowledge, the present study provides the first evidence suggesting an association between SYNE1 rs2295190 and advanced clinical stage in betel nut-exposed individuals, although the number of carriers of the variant genotype was relatively limited. Our findings highlight the importance of accounting for environmental carcinogen exposure in the assessment of the clinical significance of cancer-related genetic polymorphisms. Further studies are warranted to determine whether SYNE1 rs2295190 genotyping may have clinical utility for identifying patients at increased likelihood of presenting with advanced disease.

Our findings suggest that, although the investigated SYNE1 polymorphisms were not associated with overall OSCC susceptibility, rs2295190 may be associated with advanced clinical stage, particularly among patients with betel quid exposure.

## Figures and Tables

**Table 1:** The distributions of demographical characteristics and clinical parameters in 480 controls and 377 cases with OSCC.

Variable		Control (N=480)	Patients (N=377)	*p* Value
Age (yrs.)	>54	229 (47.7%)	196 (52.0%)	0.214
	≤54	251 (52.3%)	181 (48.0%)	
Betel nut chewing	No	372 (77.5%)	124 (32.9%)	< 0.0001*
	Yes	108 (22.5%)	253 (67.1%)	
Cigarette smoking	No	361 (75.2%)	80 (21.2%)	< 0.0001*
	Yes	119 (24.8%)	297 (78.8%)	
Alcohol drinking	No	361 (75.2%)	252 (66.8%)	0.007*
	Yes	119 (24.8%)	125 (33.2%)	
Stage	I + II		205 (54.4%)	
	III + IV		172 (45.6%)	
Tumor T status	T1 + T2		247 (65.5%)	
	T3 + T4		130 (34.5%)	
Lymph node status	N0		276 (73.2%)	
	N1 + N2 + N3		101 (26.8%)	
Metastasis	M0		357 (94.7%)	
	M1		20 (5.3%)	
Cell differentiation	Well differentiated		60 (15.9%)	
	Moderately or poorly differentiated		317 (84.1%)	

N: number. * *p* value < 0.05 as statistically significant.

**Table 2:** The distribution of genotype frequencies in SYNE1 SNPs in cases of control and OSCC group.

Variable	Control (N=480)	Patients (N=377)	OR ^a^ (95% CI)	*p* Value	AOR ^b^ (95% CI)	*p* Value
rs2295190						
GG	420 (87.5%)	343 (91.0%)	1.000 (reference)		1.000	
GT	56 (11.7%)	33 (8.8%)	0.722 (0.459-1.135)	0.158	0.828 (0.474-1.449)	0.509
TT	4 (0.8%)	1 (0.3%)	0.306 (0.034-2.752)	0.291	0.266 (0.016-4.545)	0.360
GT + TT	60 (12.5%)	34 (9.0%)	0.694 (0.445-1.082)	0.107	0.792 (0.457-1.371)	0.404
rs2306916						
TT	327 (68.1%)	276 (73.2%)	1.000 (reference)		1.000	
TA	142 (29.6%)	93 (24.7%)	0.776 (0.571-1.054)	0.105	0.897 (0.616-1.305)	0.570
AA	11 (2.3%)	8 (2.1%)	0.862 (0.342-2.172)	0.752	1.374 (0.430-4.389)	0.592
TA + AA	153 (31.9%)	101 (26.8%)	0.782 (0.581-1.054)	0.106	0.924 (0.641-1.331)	0.670
rs2695261						
AA	350 (72.9%)	269 (71.4%)	1.000 (reference)		1.000	
AG	114 (23.8%)	97 (25.7%)	1.107 (0.809-1.515)	0.525	0.785 (0.529-1.166)	0.231
GG	16 (3.3%)	11 (2.9%)	0.895 (0.408-1.959)	0.780	1.082 (0.428-2.737)	0.868
AG + GG	130 (27.1%)	108 (28.6%)	1.081 (0.800-1.460)	0.612	0.817 (0.561-1.190)	0.292
rs9371601						
TT	284 (59.2%)	229 (60.7%)	1.000 (reference)		1.000	
TG	180 (37.5%)	134 (35.5%)	0.923 (0.695-1.226)	0.581	0.973 (0.686-1.379)	0.877
GG	16 (3.3%)	14 (3.7%)	1.085 (0.519-2.270)	0.828	1.065 (0.431-2.635)	0.891
TG + GG	196 (40.8%)	148 (39.3%)	0.936 (0.711-1.233)	0.640	0.981 (0.698-1.377)	0.910

N: number. ^a^ The odds ratio (OR) with their 95% confidence intervals were estimated by logistic regression models. ^b^ The adjusted odds ratio (AOR) with their 95% confidence intervals were estimated by multiple logistic regression models after controlling for betel nut chewing, alcohol and tobacco consumption.

**Table 3:** Clinical statuses and SYNE1 rs2295190 genotype frequencies in cases of OSCC group.

Variable	SYNE1 (rs2295190)					
	GG (%) (N = 343)	GT + TT (%) (N = 34)	OR^a^ (95% CI)	*p* Value	AOR^b^ (95% CI)	*p* Value
Clinical stage						
Stage I/II	193 (56.3%)	12 (35.3%)	1.000	0.022^*^	1.000	0.030^*^
Stage III/IV	150 (43.7%)	22 (64.7%)	2.359 (1.131-4.919)		2.271 (1.081-4.771)	
Tumor size						
T1 + T2	228 (66.5%)	19 (55.9%)	1.000	0.218	1.000	0.264
T3 + T4	115 (33.5%)	15 (44.1%)	1.565 (0.767-3.194)		1.507 (0.734-3.094)	
Lymph node metastasis						
No	254 (74.1%)	22 (64.7%)	1.000	0.243	1.000	0.265
Yes	89 (25.9%)	12 (35.3%)	1.557 (0.740-3.275)		1.532 (0.723-3.246)	
Distant metastasis						
No	327 (95.3%)	30 (88.2%)	1.000	0.090	1.000	0.096
Yes	16 (4.7%)	4 (11.8%)	2.725 (0.856-8.673)		2.722 (0.838-8.844)	
Cell differentiation						
Well	53 (15.5%)	7 (20.6%)	1.000	0.437	1.000	0.544
Moderate/ poor	290 (84.5%)	27 (79.4%)	0.705 (0.292-1.702)		0.759 (0.311-1.853)	

N: number. ^a^ The odds ratio (OR) with their 95% confidence intervals were estimated by logistic regression models. ^b^ The adjusted odds ratio (AOR) with their 95% confidence intervals were estimated by multiple logistic regression models after controlling for betel nut chewing, alcohol and tobacco consumption. * *p* value < 0.05 as statistically significant.

**Table 4:** The association between SYNE1 rs2295190 genotype frequency and clinical status with and without betel nut chewers.

Variable	SYNE1 (rs2295190)					
	With betel nut chewing (N=253)			Without betel nut chewing (N=124)		
	GG (%) (N =227)	GT + TT (%) (N =26)	*p* Value	GG (%) (N =116)	GT + TT (%) (N =8)	*p* Value
Clinical stage						
Stage I/II	127 (55.9%)	9 (34.6%)	0.043^*^	66 (56.9%)	3 (37.5%)	0.296
Stage III/IV	100 (44.1%)	17 (65.4%)		50 (43.1%)	5 (62.9%)	
Tumor size						
T1 + T2	148 (65.2%)	15 (57.7%)	0.450	80 (69.0%)	4 (50.0%)	0.277
T3 + T4	79 (34.8%)	11 (42.3%)		36 (31.0%)	4 (50.0%)	
Lymph node metastasis						
No	169 (74.4%)	17 (65.4%)	0.324	85 (73.3%)	5 (62.5%)	0.512
Yes	58 (25.6%)	9 (34.6%)		31 (26.7%)	3 (37.5%)	
Distant metastasis						
No	215 (94.7%)	22 (84.6%)	0.056	112 (96.6%)	8 (100.0%)	0.999
Yes	12 (5.3%)	4 (15.4%)		4 (3.4%)	0 (0.0%)	
Cell differentiation						
Well	39 (17.2%)	6 (23.1%)	0.458	14 (12.1%)	1 (12.5%)	0.971
Moderate/poor	188 (82.8%)	20 (76.9%)		102 (87.9%)	7 (87.5%)	

N: number. * *p* value < 0.05 as statistically significant. OR (95% CI): 2.399 (1.026 - 5.609)

**Table 5:** Clinical status and SYNE1 rs2295190 genotype frequency in OSCC cases classified by betel quid additives.

Variable	SYNE1 (rs2295190)								
	Areca fruit with slaked lime and piper betel leaf (N=137)			Areca fruit with slaked lime and piper betel inflorescence (N=62)			Consumed with two betel nut additives (N=48)		
	GG (%)(N = 121)	GT + TT (%)(N = 16)	*p* Value	GG (%)(N = 58)	GT + TT (%)(N = 4)	*p* Value	GG (%)(N = 42)	GT + TT (%)(N = 6)	*p* Value
Clinical stage									
Stage I/II	65 (53.7%)	4 (25.0%)	*p* = 0.039^*^	32 (55.2%)	2 (50.0%)	*p* = 0.841	27 (64.3%)	3 (50.0%)	p = 0.503
Stage III/IV	56 (46.3%)	12 (75.0%)		26 (44.8%)	2 (50.0%)		15 (35.7%)	3 (50.0%)	
Tumor size									
T1 + T2	78 (64.5%)	8 (50.0%)	*p* = 0.266	37 (63.8%)	3 (75.0%)	*p* = 0.654	30 (71.4%)	4 (66.7%)	p = 0.811
T3 + T4	43 (35.5%)	8 (50.0%)		21 (36.2%)	1 (25.0%)		12 (28.6%)	2 (33.3%)	
Lymph node metastasis									
No	84 (69.4%)	9 (56.3%)	*p* = 0.293	45 (77.6%)	3 (75.0%)	*p* = 0.905	34 (81.0%)	5 (83.3%)	p = 0.889
Yes	37 (30.6%)	7 (43.8%)		13 (22.4%)	1 (25.0%)		8 (19.0%)	1 (16.7%)	
Distant metastasis									
No	114 (94.2%)	13 (81.3%)	*p* = 0.077	57 (98.3%)	4 (100.0%)	*p* = 1.000	39 (92.9%)	5 (83.3%)	p = 0.444
Yes	7 (5.8%)	3 (18.7%)		1 (1.7%)	0 (0.0%)		3 (7.1%)	1 (16.7%)	
Cell differentiation									
Well	22 (18.2%)	3 (18.8%)	*p* = 0.956	10 (17.2%)	0 (0.0%)	*p* = 1.000	6 (14.3%)	3 (50.0%)	p = 0.054
Moderate/poor	99 (81.8%)	13 (81.3%)		48 (82.8%)	4 (100.0%)		36 (85.7%)	3 (50.0%)	

N: number. * *p* value < 0.05 as statistically significant. OR (95% CI): 3.482 (1.063 - 11.408)

## Data Availability

The data used to support this study's findings are available from the corresponding author upon request.
